# Larvicidal Activity of Essential Oils From *Piper* Species Against Strains of *Aedes aegypti* (Diptera: Culicidae) Resistant to Pyrethroids

**DOI:** 10.3389/fpls.2021.685864

**Published:** 2021-06-04

**Authors:** Adalberto Alves Pereira Filho, Grasielle C. D‘Ávila Pessoa, Lydia F. Yamaguchi, Mariana Alves Stanton, Artur M. Serravite, Rafael H. M. Pereira, Welber S. Neves, Massuo Jorge Kato

**Affiliations:** ^1^Laboratório de Fisiologia de Insetos Hematófagos, Departamento de Parasitologia/ICB, Universidade Federal de Minas Gerais, Belo Horizonte, Brazil; ^2^Laboratory of Natural Product Chemistry, Department of Fundamental Chemistry, University of São Paulo, São Paulo, Brazil

**Keywords:** essential oils, *Piper*, larvicides, vector control, *Aedes aegypti*, insecticide resistance

## Abstract

The continuous and indiscriminate use of insecticides has been responsible for the emergence of insecticide resistant vector insect populations, especially in *Aedes aegypti*. Thus, it is urgent to find natural insecticide compounds with novel mode of action for vector control. The goal of this study was to investigate the larvicidal activity of essential oils (EOs) from *Piper* species against *A. aegypti* characterized as resistant and susceptible strains to pyrethroids. The EOs from leaves of 10 *Piper* species were submitted to the evaluation of larvicidal activity in populations of *A. aegypti* in agreement with the (World Health Organization, 2005) guidelines. The resistance of the strains characterized by determining the lethal concentrations (LCs) with the insecticide deltamethrin (positive control). The major compounds of the EOs from *Piper* species was identified by GC-MS. The EOs from *Piper aduncum, P. marginatum, P. gaudichaudianum, P. crassinervium*, and *P. arboreum* showed activity of up to 90% lethality at 100 ppm (concentration for screening). The activities of the EOs from these 6 species showed similar LCs in both susceptible strain (Rockefeller) and resistant strains (Pampulha and Venda Nova) to pyrethroids. The major compounds identified in the most active EO were available commercially and included β-Asarone, (*E*)-Anethole, (*E*)-β-Caryophyllene, γ-Terpinene, *p*-Cymene, Limonene, α-Pinene, and β-Pinene. Dillapiole was purified by from EO of *P. aduncum*. The phenylpropanoids [Dillapiole, (*E*)-Anethole and β-Asarone] and monoterpenes (γ-Terpinene, *p*-Cymene, Limonene, α-Pinene, and β-Pinene) showed larvicidal activity with mortality between 90 and 100% and could account for the toxicity of these EOs, but the sesquiterpene (*E*)-β-Caryophyllene, an abundant component in the EOs of *P. hemmendorffii* and *P. crassinervium*, did not show activity on the three populations of *A. aegypti* larvae at a concentration of 100 ppm. These results indicate that *Piper'*s EOs should be further evaluated as a potential larvicide, against strains resistant to currently used pesticides, and the identification of phenylpropanoids and monoterpenes as the active compounds open the possibility to study their mechanism of action.

## Introduction

*Aedes aegypti* (Linnaeus, 1762) (Mattingly et al., 1962) is a mosquito species known to transmit arboviruses such as dengue, chikungunya, and zika virus worldwide. It is a diurnal mosquito extremely adapted to urban and domestic environments (Maciel-de-Freitas et al., 2012). The rapid increase in rates of urbanization in tropical regions, lack of basic infrastructure and limited or non-existent sanitation, associated with favorable climatic conditions for the mosquito's development, have contributed to the expansion of the occurrence range of arboviruses transmitted by females of *A. aegypti* (Rebêlo et al., 1999; Carvalho and Moreira, 2016). Therefore, we have witnessed the increasing transmission of Dengue (DENV), Zika (ZIKV), and Chikungunya (CHIKV) virus in these regions.

Despite the FDA approval of a dengue vaccine (Dengvaxia) in 2019, its efficiency is restricted to people who have been previously infected by dengue and not as disease prevention for a large portion of the population. Therefore, the control of *A. aegypti* populations still represents the best line of defense. This strategy has focused on controlling the mosquito's population by means of using insecticides such as the larvicide Pyriproxyfen (Juvenil Hormone Analog–JHA), the adulticides malathion (organophosphate) and Cielo®, an insecticide containing imidacloprid (neonicotinoid) and paletrine (pyrethroid) (Valle et al., 2019). The main larvicide used worldwide was the organophosphate temephos, but by the end of 1990's, it led to the development of resistance in *A. aegypti*. In fact, in the last decades, the indiscriminate use of synthetic insecticides (for example, domestic use of pyrethroid insecticides available in the retail market, especially in epidemic periods), together with the lack of coordinated programs in multi-endemic areas, have led to the emergence of populations of *A. aegypti* resistant to different insecticides used (Maciel-de-Freitas et al., 2012; Macoris et al., 2018).

The resistance of *A. aegypti* in Brazil studied from 2005 to 2012 was characterized by the frequency and distribution of the resistance of this vector (Valle et al., 2019). The phenotypes for populations resistant to pyrethroids throughout the country have been characterized and are associated to the changes in biochemical and target site mutations V410L, G923V, I1011M, V1016I, and F1534C (Brengues et al., 2003; Saavedra-Rodriguez et al., 2007; Martins et al., 2009a,b; Lima et al., 2011; Araújo et al., 2013; Lins et al., 2014; Maciel-de-Freitas et al., 2014; Bellinato et al., 2016; Collet et al., 2016; Dolabella et al., 2016; Haddi et al., 2017; Viana-Medeiros et al., 2017; Garcia et al., 2018; Valle et al., 2019; Costa et al., 2020).

In the case of larval resistance in *A. aegypti*, two studies were carried out in the city of Belo Horizonte (MG, Brazil) (Belinato et al., 2013; Valle et al., 2019). The resistance ratio 95 (RR_95_) to the insecticide Temephos was quantified in mosquito populations in 2005 (Belinato et al., 2013) and 2008 (Valle et al., 2019), in which resistance was observed (RR_95_ = 5.4 and RR_95_ = 10.8, respectively).

Essential oils (EOs) are odoriferous and volatile compounds found stored in plants structures, such as glands, secretory trichomes, secretory ducts, secretory cavities, or resin ducts (Ciccarelli et al., 2008; Bezić et al., 2009; Liolios et al., 2010; Morone-Fortunato et al., 2010). The production of these volatiles in plants is associated with the ecological role they display in nature, such as protecting plants against pathogens and herbivores and attraction of pollinating insects (Grodnitzky and Coats, 2002; Csekea et al., 2007; Bakkali et al., 2008). The emission of plant volatiles is associated with several messages they convey to the surrounding interacting organisms, such as volatiles used in the attraction of pollinating insects, kairomones in response to herbivores, attraction of parasitoids when damaged by herbivores, controlling the growth of pathogens in aerial parts or roots, and so forth (Grodnitzky and Coats, 2002; Csekea et al., 2007; Bakkali et al., 2008; Raveau et al., 2020). EOs from the plants *Cymbopogon* spp., *Ocimum* spp. and *Eucalyptus* spp. are well-known for their application as insect repellents and the active principles are associated with the presence of α-Pinene, Limonene, Citronellol, Citronellal, Camphor and Thymol (Nerio et al., 2010). Additionally, some plant species have been adopted in push-pull strategies for controlling insect pests in agricultures thanks to their emission of specific volatile compounds with repellent or attractive properties that lead pests away from cultivated plants and onto toxic “trap crops” (Cook et al., 2007; Alkema et al., 2019).

Several applications of volatiles of *Piper* species have been suggested because of their high potential for pest control, and due to the green technologies involved in the extraction process and the expected low environmental impact (da Silva et al., 2017; Salehi et al., 2019). The evaluation of the larvicidal activity of EOs of species of the genus *Piper* in *A. aegypti* has already been studied for the species: *Piper humaytanum, P. permucronatum, P. hostmanianum, P. gaudichaudianum* (de Morais et al., 2007), *P. augustum, P. corrugatum, P. curtispicum, P. darienense, P. grande, P. hispidum, P. jacquemontianum, P. longispicum, P. multiplinervium, P. reticulatum, P. trigonum* (Santana et al., 2016), *P. marginatum* (Autran et al., 2009; Santana et al., 2015), *P. klotzschianum* (Nascimento et al., 2013), *P. aduncum* (de Almeida et al., 2009; Oliveira et al., 2013; Santana et al., 2015; Scalvenzi et al., 2019), *P. corcovadensis* (da Silva et al., 2016), *P. sarmentosum* (Hematpoor et al., 2016), *P. betle* (Vasantha-Srinivasan et al., 2018; Martianasari and Hamid, 2019), *P. arboreum* (Santana et al., 2015), and *P. capitarianum* (França et al., 2021). Besides, non-volatile compounds from *Piper* species such as amides and lignans have also been described as larvicidal (Cabral et al., 2009; Kanis et al., 2018).

Despite the large number of studies with EOs from *Piper* species against larvae of *A. aegypti*, there is no assessment of their effect on strains of mosquitoes resistant to synthetic insecticides. Therefore, considering the limited number of safe chemical approaches for controlling *A. aegypti* as vectors in the field, the aim of this work is to investigate the larvicidal activity of essential oils of *Piper* species and to identify the active principle against populations of *A. aegypti* that are either susceptible or resistant to pyrethroids.

## Materials and Methods

### Plant Material

The leaves of 10 species of plants belonging to the genus *Piper: Piper aduncum, P. marginatum, P. gaudichaudianum, P. crassinervium, P. arboreum, P. hemmendorffii, P. cernuum, P. lucaenum* var. *gradifolium, P. lindbergii*, and *P. amalago*, were collected in the period of January to June 2018. The vouchers were deposited at the Herbarium of USP–University of São Paulo for identification (Table 1). All collections were made under permits #59161-1 and 010/2018-R from the Sistema de Autorização e Informação em Biodiversidade–SISBIO and Fundação Serra do Japi, respectively.

**Table 1:** Voucher number, sampling site, and yield of essential oil from *Piper* species.

**Species**	**Voucher ^#^**	**Sites**	**EO yield (%)^**a**^**
*P. aduncum* L.	K-0057	Campus–USP^b^	2.5
*P. marginatum* Jacq.	K-0223	Campus–USP	1.10
*P. gaudichaudianum* Kunth	K-0031	Serra do Japi^c^	0.98
*P. crassinervium* H.B.and K.	K-0091	Campus–USP	1.80
*P. arboreum* Aubl.	K-0053	Campus–USP	0.74
*P. hemmendorffii* C. DC.	K-1086	Campus–USP	0.86
*P. cernuum* Vell.	K-0137	Campus–USP	0.69
*P. lucaenum* var. *grandifolium* Kunth.	K-0486	Campus–USP	0.70
*P. lindbergii* C.DC.	K-2325	Serra do Japi	0.65
*P. amalago* L.	K-0110	Serra do Japi	0.66

# *K (Voucher number Kato-XXXX)*.

a *From fresh leaves*.

b *University of São Paulo*.

c *Parque Municipal Serra do Japi, Jundiaí, Brazil*.

### Collection and Insect Rearing

In this study, three strains of *A. aegypti* larvae were used. The Rockefeller strain is a susceptible reference lineage (SRL) for all assays. Two other strains were collected in the regions of Pampulha (19° 51′ 04″ S; 43° 58′ 46″ W) and Venda Nova (20° 11′ 51″ S; 44° 1′ 40″ W) in Belo Horizonte, Minas Gerais, Brazil in the period of June 2018. The strains Pampulha (Pamp) and Venda Nova (VN) were evaluated and certified as resistant to pyrethroids.

The mosquitoes were kept and raised in the insectarium of the Laboratory of Physiology of Hematophagous Insects of the Federal University of Minas Gerais in accordance with the recommendations of the Ethics Committee (CEUA-UFMG) (protocol number 01/2017). The insects were maintained under controlled conditions of temperature (27 ± 1°C), photoperiod 12:12 h (L:D), and relative humidity (75%).

After the eggs hatched in dechlorinated water, the larvae and pupae were kept in plastic vats, containing fish food *ad libitum*. The adult insects were kept in cylindrical cages 30 × 90 cm with mesh on the top and with continuous access to cotton soaked in 10% sucrose solution. The females' blood meals were performed weekly on hamsters (*Mesocricetus auratus*) previously anesthetized with 0.2 mL of Thiopental® (50 mg/mL) and placed with the trichotomized abdomen on the screen of the cages so that the females could perform the blood meal for 1 h. The eggs were obtained 2 days after the meal using filter paper soaked in dechlorinated water in dark plastic pots, from which they were removed and kept in new plastic pots until the hatching time for testing.

### Extraction of Essential Oils

The essential oils (EOs) were extracted from fresh leaves of each species, submitted to hydrodistillation in a Clevenger type apparatus for 4 h, using 300–500 g of fresh leaves and 500 mL of distilled water (Santos et al., 2012; Fanela et al., 2015). The EOs were collected and dried with anhydrous sodium sulfate and stored in amber bottles in a refrigerator at 4°C until the experiments were performed. The yield of EOs from *Piper* species are shown in Table 1.

### Analysis of Essential Oils and Fractions by GC-MS

EOs samples were diluted 20 times in ethyl acetate (HPLC grade, Honeywell) and analyzed using a Shimadzu GCMS- QP2010 equipped with an HP-5ms column (length 30 m, ID 0.25 mm, film thickness 0.25 μm, Agilent) using Helium as a carrier gas (1.55 mL/min) and 1 μL of each sample was injected at 250°C with a 1:20 split. Detector temperature was set at 260°C with electron impact ionization energy of 70 eV and a scan range of *m/z*35-400 Da at 2500 spectra s^−1^. The oven program started at 40°C for 2 min, and the temperature was increased at 5°C min^−1^ to 260°C and held for 2 min. Individual volatile compound peaks were identified using extracted ion traces of three specific reference ions and quantified by the peak area of the most abundant ion trace per compound using a custom-made analysis method in the GC-MS Postrun Analysis software (Shimadzu). Relative % of each compound was calculated by comparing the % peak area in relation to the total sum of peak areas within a sample. The identification of compounds was conducted by calculating Arithmetic retention indexes (RIs) in relation to a series of alkane standards (C8-C40, Supelco) injected using the same GC-MS method as used for the samples, according to Van Den Dool and Kratz (1963). Compound mass spectra and RIs were compared to those available in the Adams and Wiley databases (Adams, 2007), in previous studies of these *Piper* species, and confirmed by comparison to authentic standards, when available.

### LC_50_ and LC_95_ for Larvicidal Activity of Deltamethrin Insecticide

The characterization of the larvae to be used in the tests as susceptible or resistant was made by assaying them with the technical grade insecticide deltamethrin (Bayer Brazil, 99.1%). The dose-response tests were performed in the range of 10–90% mortality. Thirty L3-L4 larvae (F1 generation) were separated per dose (in triplicate), requiring a minimum of 8 doses to perform the curve. The larvae were placed in 500 ml cups containing 249 ml of dechlorinated water, along with 1 ml of the insecticide in the desired concentration, diluted in ethanol P.A. For the control group, 30 L3-L4 larvae were used in 250 mL of dechlorinated water. The concentrations of pyrethroid used for assaying Rockefeller, Venda Nova and Pampulha strains were 0.2–0, 9, 2.0–9.5, and 1.0–8.0 mg/mL, respectively.

Mortality was recorded every 10 min during the first hour and 24 h after the start of the test, as recommended by the World Health Organization (1981). Larvae that did not spontaneously move, even if subjected to mechanical stimulation, were considered dead. The LC calculation was performed using the Polo Plus program (see item on statistical analysis). The LC of the field population was divided by the LC of the Rockefeller strain to obtain the Resistance Ratio. The population was considered resistant when RR_95_ was >3 (Valle et al., 2019).

### Qualitative Larvicidal Bioassays of *Piper* Essential Oils

The tests were performed in accordance with World Health Organization (2005), with modifications. The EOs of 10 species of *Piper* were diluted in a final volume of 100 ml of dechlorinated water with 2% dimethyl sulfoxide (DMSO), in a concentration of 100 ppm of each oil. Then, 30 larvae (L3–L4) from the three strains, Rockefeller, Venda Nova, and Pampulha were placed in the containers. Each experiment was carried out in three bottles (technical triplicate), being repeated five times on different days (five biological repetitions). The larvae of the control and vehicle control groups were exposed only to dechlorinated water and with 2% DMSO, respectively. Mortality was recorded at 24 h after the start of the test. Larvae not responding to mechanical stimulation were considered dead and the EOs with 90 to 100% larvicidal activity were considered active (Cheng et al., 2003; Dias et al., 2014; Intirach et al., 2016; Muturi et al., 2017).

### Determination of LC_50_ and LC_90_ of *Piper* Essential Oils

EOs that showed preliminary larvicidal activity (90 to 100%) had their lethal concentrations (LCs) determined. Thus, 30 larvae L3-L4 from the three strains were submitted to different concentrations in a range of 10–90%, in a final volume of 100 mL of dechlorinated water. Each dose was assayed in duplicate, with three repetitions (biological triplicate) on different days. The larvae of the control and vehicle-control groups were exposed to dechlorinated water and with 2% DMSO, respectively, and the mortality was recorded 24 h after the start of the test.

### Assays With the Major Compounds From the EOs

The major compounds from EOs characterized by GC-MS, *E*-Anethole (Sigma-Aldrich: 4180-23-8), γ-Asarone (Cayman Chemical: 11681) and (*E*)-β-Caryophyllene (Cayman Chemical: 21572), γ-Terpinene (Sigma-Aldrich: 86478), p-Cymene (Sigma-Aldrich: 121452), Limonene (Sigma-Aldrich: 45423), α-Pinene (Sigma-Aldrich: 147524), and β-Pinene (Sigma-Aldrich: 402753) were acquired commercially. Pure Dillapiole was obtained by fractionation using the Isolera Flash Chromatography system (Biotage INC). The EO of *P. aduncum* (0.5 ml) was loaded on the silica samplet and the flash chromatography was performed in the SNAP Ultra 25 g silica column using a gradient of hexane and ethyl acetate. The gradient started with 20% of ethyl acetate, after 2 min increased to 28% and in a linear increase reached 33% 12 min. Sixty fractions were collected and dillapiole was present in the fractions 30–35. The samples obtained from this fractionation were analyzed by GC-MS and a fraction with purity higher than 98% was selected for the assays.

For the larvicidal assays (qualitative bioassays), the pure compounds were diluted in dechlorinated water and 2% DMSO, in a final volume of 50 mL, to a final concentration of 100 ppm (screening concentration), containing 15 larvae of *A. aegypti* L3-L4 per cup. For the determination of LC_50_ and LC_90_, the methodology was used as described above, using 15 larvae of *A. aegypti* L3-L4 per cup.

### Statistical Analysis

The data were organized in spreadsheets using Microsoft Excel software (Office 2007). Lethal concentrations (LC) 50%, 90%, 95% and slope were obtained through Probit analysis with the aid of the Polo Plus software (Raymond, 1985). Significant differences in the LC_50_ and LC_90_ values were based on non-overlap of 95% confidence intervals (Hematpoor et al., 2017; Wang et al., 2019).

## Results

### Determination of LC_50_ and LC_95_ for the Insecticide Deltamethrin

Based on the bioassays with Deltamethrin, the strains of Pampulha and Venda Nova were shown to be resistant to this insecticide, with the population of Pampulha (RR_95_ = 26.073) being more resistant than the population of Venda Nova (RR_95_ = 20.512). The resistance observed in these populations of *A. aegypti* for Deltamethrin is expected for pyrethroids in general, because of the similarity of the mode of action. The Rockefeller strain, defined as the susceptibility reference strain (LRS), has been maintained in the laboratory since 1881, without contact with insecticides and genetically isolated from external populations (Organização Pan-americana de Saúde (OPAS), 2005). The values of LC_50_ and LC_95_ with their 95% confidence intervals are listed in Table 2.

**Table 2:** LC_50_ and LC_95_ for the technical grade deltamethrin insecticide (Bayer Brazil, 99.1%) in larvae of *Aedes aegypti*.

**Strains**	**LC_**50**_ (mg/L) (95% CI)**	**RR_**50**_ (95% CI)**	**LC_**95**_ (mg/L) (95% CI)**	**RR_**95**_ (95% CI)**	**Slope (SD)**
Rockefeller^*^	0.16 (0.14–0.189)	1	0.751 (0.55–1.22)	1	3.55 ± 0.45
Venda Nova	3.65 (3.21–4.08)	21.94 (18.36–26.23)	9.905 (8.29–12.80)	20.51 (14.44–29.12)	3.79 ± 0.41
Pampulha	2.94 (2.42–3.47)	17.67 (14.17–22.02)	12.590 (9.20–21.13)	26.07 (16.07–42.28)	2.60 ± 0.36

*95% CI, 95% confidence interval; LC_50_, 50% lethal concentration; LC_95_, 95% lethal concentration; RR_95_, 95% resistance ratio; SD, standard deviation*.

* *SRL—susceptibility reference lineage*.

### Larvicidal Activity of Essential Oils

The EOs of the leaves of 10 species of the genus *Piper* obtained from hydrodistillation were tested against the three strains (resistant and susceptible) of *A. aegypti*. EOs from 5 out of 10 species were considered active: *Piper aduncum, P. marginatum, P. gaudichaudianum, P. crassinervium*, and *P. arboreum*, with larvicidal activity of 90–100% at 100 ppm (Table 3).

**Table 3:** Mortality percentage of *Aedes aegypti* larvae in resistant and susceptible strains to pyrethroids treated with essential oils of *Piper* species.

**Species**	**Strains**		
	**Susceptible**	**Resistant**	
	**Rockefeller**	**Venda Nova**	**Pampulha**
*P. aduncum* L.	100.00	100.00	100.00
*P. marginatum* Jacq.	100.00	97.11	98.44
*P. gaudichaudianum* Kunth	99.33	90.22	94.88
*P. crassinervium* H.B. and K.	96.22	91.11	92.66
*P. arboreum* Aubl.	93.11	90.66	90.00
*P. hemmendorffii* C. DC.	66.44	43.11	36.00
*P. cernuum* Vell.	45.11	42.00	30.44
*P. lucaenum* Kunth.	16.22	18.88	24.00
*P. lindbergii* C. DC.	12.00	13.55	10.22
*P. amalago* L.	2.66	6.22	2.22

EOs of these five species had their lethal concentrations, LC_50_ and LC_90_ investigated. Thus, the L3-L4 larvae of the two populations and LRS were submitted to different concentrations to achieve larval mortality in a range of 10–90%. After 24 h of exposure the EOs from *P. aduncum, P. gaudichaudianum*, and *P. marginatum* had the lowest LC_50_ compared to *P. crassinervium* and *P. arboreum*. The EOs from *P. aduncum* was the most active with LC_50_ (23.50 ppm) for Rockefeller, and LC_50_ of 25.11 ppm and 26.39 ppm to Venda Nova and Pampulha, respectively (Table 4).

**Table 4:** Lethal concentrations of essential oil of *Piper* species against *Aedes aegypti* larvae resistant and susceptible strains to pyrethroids, during 24 h of exposure.

**Species**	**Strains**	**Slope ± SD**	**LC_**50**_ (ppm) (95% CI)**	**LC_**90**_ (ppm) (95% CI)**
*P. aduncum*	Rockefeller	4.5 ± 0.2	23.50 (20.92–26.60)	45.25 (37.46–62.61)
	Venda Nova	4.2 ± 0.2	25.11 (22.92−27.80)	50.29 (42.37–65.56)
	Pampulha	4.3 ± 0.2	26.39 (24.69–28.40)	52.08 (45.58–62.60)
*P. gaudichaudianum*	Rockefeller	4.3 ± 0.2	37.88 (29.58–46.21)	75.20 (58.19–142.75)
	Venda Nova	5.6 ± 0.3	54.01 (49.50–58.95)	91.41 (79.56–115.2)
	Pampulha	3.9 ± 0.2	41.35 (34.57–49.01)	86.61 (67.51–148.57)
*P. marginatum*	Rockefeller	4.1 ± 0.2	39.91 (34.94–45.11)	80.85 (66.94–112.42)
	Venda Nova	5.0 ± 0.2	41.72 (37.05–46.80)	87.27 (72.22–120.23)
	Pampulha	4.0 ± 0.2	45.77 (43.65–48.05)	96.06 (87.07–108.70)
*P. arboreum*	Rockefeller	5.4 ± 0.3	51.63 (47.68–55.72)	89.13 (78.69–108.22)
	Venda Nova	5.6 ± 0.3	54.01 (49.50–58.95)	91.41 (79.56–115.27)
	Pampulha	5.5 ± 0.3	56.22 (52.16–60.82)	96.01 (84.15–118.36)
*P. crassinervium*	Rockefeller	4.9 ± 0.3	59.03 (53.36–66.47)	106.81 (88.43–152.98)
	Venda Nova	5.1 ± 0.3	63.55 (58.34–70.79)	113.21 (95.01–154.04)
	Pampulha	5.1 ± 0.3	62.96 (57.94–69.77)	112.10 (94.58–150.34)

*95% CI, 95% confidence interval; LC50, 50% lethal concentration; LC90, 90% lethal concentration; SD, standard deviation*.

### Identification of Essential Oil Compounds by GC-MS

The EOs of the 10 species were analyzed by GC-MS and main constituents were identified based on library search, retention index (RI), and use of standard compounds when available, and expressed as relative percentage of each constituent (Table 5). In summary, the major compounds were identified as phenylpropanoids, sesquiterpenes and monoterpenes. The complete list of all compounds, the retention indexes, and the relative percentage of each one, for all 10 species of *Piper* analyzed is shown in Table 6 and the GC-MS chromatograms for all species is shown in the Supplementary Figures 1–3.

**Table 5:** Major constituents of the EOs of *Piper* species.

**Species**	**Major compounds (class)^**a**^**	**RI**	**% rel**.
*P. aduncum*	Dillapiole (P)	1632	81.01
*P. arboreum*	Germacrene D (S)	1484	18.58
	δ-Elemene (S)	1339	14.53
*P. crassinervium*	α-Pinene (M)	932	13.95
	β-Pinene (M)	975	12.09
	(*E*)-β-Caryophyllene (S)	1422	8.01
*P. gaudichaudianum*	α-Humulene (S)	1457	15.50
	Bicyclogermacrene (S)	1500	13.53
*P. marginatum*	(*E*)*-*Isoosmorhizole (P)	1462	35.23
	(*E*)*-*Anethole (P)	1286	21.67
*P. hemmendorffii*	Limonene (M)	1039	30.99
	β-Pinene (M)	975	10.08
	(*E*)-β-Caryophyllene (S)	1422	9.65
*P. amalago*	α-Pinene (M)	932	28.80
	(*E*)-Nerolidol (S)	1566	9.2
	*p*-Cymene (M)	1024	8.4
*P. lindbergii*	α-Pinene (M)	932	61.67
	α-Copaene (S)	1378	6.4
	Limonene (M)	1039	5.3
*P. cernuum*	α-Pinene (M)	932	16.6
	β-Pinene (M)	975	11.5
	Bicyclogermacrene (S)	1500	10.7
*P. lucaenum*	Bicyclogermacrene (S)	1500	27.47
	(*E*)-Cadina-1,4-diene (S)	1527	21
	β-Myrcene (M)	992	10.7

*RI, retention index; % rel., relative percentage*.

a *P, Phenylpropanoid; S, Sesquiterpene; M, Monoterpene*.

**Table 6:** Chemical composition of the essential oils of *Piper* species.

**Compounds**	**RI^**a**^**	**RI^**b**^**	**PAD**	**PAR**	**PAB**	**PCR**	**PGA**	**PAM**	**PHE**	**PLB**	**PAP**	**PCE**	**PLU**
α-Pinene^S^	932	932	0.2	1.0	1.5	14.0	–	0.8	3.2	28.8	61.7	16.6	–
Camphene^S^	947	946	–	–	–	–	–	0.4	–	–	1.7	0.1	2.2
β-Pinene^S^	975	974	0.3	0.6	–	12.1	–	0.7	10.1	3.0	1.4	11.5	–
Sulcatone	989	981	–	–	–	6.2	–	–	–	–	–	–	–
β-Myrcene^S^	992	988	–	1.7	–	0.6	–	–	0.7	5.9	0.3	1.0	10.7
α-Phellandrene^S^	1,004	1,002	0.1	0.5	3.7	0.5	–	–	–	0.7	–	0.2	1.4
2-Carene^S^	1,010	1,008	0.1	0.7	–	1.5	–	0.8	0.5	0.7	–	0.2	0.1
α-Terpinene	1,016	1,014	–	6.8	–	–	–	–	–	–	–	4.5	–
*p*-Cymene^S^	1,024	1,020	0.1	3.0	1.5	0.5	–	–	0.3	8.4	1.0	9.2	–
Limonene^S^	1,039	1,024	0.1	–	2.1	1.2	0.2	0.1	30.9	–	5.3	0.8	1.9
(*Z*)-β-Ocimene^S^	1,039	1,032	1.6	–	8.5	0.1	0.5	0.1	0.3	2.1	–	0.1	4.6
(*E*)-β-Ocimene^S^	1,049	1,044	3.4	–	4.9	0.1	0.7	0.2	3.2	0.3	–	0.3	–
γ-Terpinene	1,059	1,054	0.2	22.6	–	0.1	–	–	–	–	–	9.9	0.3
α-Terpinolene^S^	1,088	1,086	0.4	11.5	–	0.1	–	–	0.1	–	–	2.7	0.2
2-Nonanone	1,092	1,087	–	3.1	–	–	–	–	–	–	–	–	–
Linalool^S^	1,100	1,095	–	–	0.6	2.4	0.7	–	–	2.0	1.6	–	5.9
(*E*)-4,8-dimethyl-1,3,7-nonatriene (DMNT)^S^	1,117	1,114	–	–	1.1	0.7	–	–	0.6	–	–	0.3	–
Camphor^S^	1,144	1,141	–	–	–	–	–	0.2	–	–	1.1	–	–
Terpinen-4-ol	1,178	1,174	–	–	–	–	–	–	–	–	0.1	0.3	1.0
α-Terpineole	1,191	1,186	–	–	–	0.6	0.4	–	0.7	–	2.3	0.3	1.1
Methyl-chavicol	1,198	1,195	–	–	–	–	–	0.2	–	–	–	–	–
Oxygenated monoterpene 1^*^	1,201	–	–	–	–	–	0.1	–	–	–	–	–	–
Oxygenated monoterpene 2^*^	1,209	–	0.1	–	–	–	0.3	–	–	–	0.1	–	–
Verbenone	1,209	1,204	–	–	–	–	–	–	–	–	–	–	–
(*Z*)-Anethole	1,253	1,249	–	–	–	–	–	6.1	–	–	–	–	–
(+)-Piperitone	1,255	1,249	0.7	–	–	–	–	–	–	–	–	–	–
(*E*)-anethole^S^	1,286	1,282	–	–	–	–	–	21.7	–	–	–	–	0.1
Safrole^S^	1,289	1,285	–	4.1	–	–	1.4	–	–	–	–	–	–
δ-Elemene	1,339	1,335	0.1	0.5	14.5	2.1	0.6	–	0.1	1.7	1.1	0.4	1.5
α-Cubebene	1,352	1,345	–	–	0.3	0.7	0.7	–	0.3	–	0.1	0.3	1.7
α-Ylanglene	1,374	1,373	0.1	–	–	1.1	0.4	–	0.1	–	–	–	–
α-Copaene^S^	1,378	1,374	0.2	–	1.4	2.9	2.8	1.4	4.1	0.2	6.4	2.1	0.7
β-Bourbonene	1,387	1,387	–	0.5	0.3	0.5	0.1	0.2	–	2.2	0.2	0.6	2.4
β-Elemene	1,394	1,389	0.2	0.2	1.4	1.0	1.8	0.5	0.6	3.3	0.3	4.4	–
(*E*)-Caryophyllene	1,404	1,408	–	–	–	–	–	–	0.5	–	–	–	–
Methyl-eugenol^S^	1,406	1,403	–	4.0	–	–	–	0.1	–	–	–	–	–
α-Gurjunene^S^	1,412	1,409	0.1	–	–	0.3	0.7	–	0.2	1.0	–	0.1	0.2
(*E*)-β-Caryophyllene^S^	1,422	1,417	0.8	0.3	8.1	8.0	4.8	2.7	9.6	0.1	0.5	7.0	2.7
β-Gurjunene	1,432	1,431	0.2	0.2	1.0	2.2	1.4	0.2	0.9	4.1	0.6	0.5	0.3
α-Guaiene	1,436	1,437	–	–	0.4	0.2	0.8	–	–	0.8	–	–	0.2
(+)-Aromadendrene^S^	1,442	1,439	–	–	0.5	1.5	4.4	–	0.4	0.3	0.2	0.4	0.9
α-Himachalene	1,446	1,449	–	–	–	–	0.8	–	–	–	–	–	0.1
α-Humulene^S^	1,457	1,452	0.9	–	4.9	2.8	15.5	0.9	2.7	1.2	0.2	2.1	0.5
Isoosmorhizole^±^	1,462	1,466	–	–	–	–	–	15.4	–	–	–	–	–
Croweacin	1,463	1,457	1.1	10.4	–	–	–	–	–	–	–	–	–
(-)-Alloaromadendrene^S^	1,464	1,458	–	–	–	0.4	–	–	0.5	0.5	0.9	0.1	0.3
Dehydro-aromadendrane	1,466	1,460	–	–	–	0.4	1.6	–	–	1.9	0.9	–	0.3
γ-Muurolene	1,480	1,479	–	–	2.1	–	2.8	0.1	1.3	0.8	1.3	0.5	0.2
Germacrene D	1,484	1,481	2.7	2.1	18.6	–	4.0	0.1	2.7	2.8	–	5.2	3.4
β-Selinene	1,490	1,490	–	–	–	–	1.2	1.1	1.0	–	0.3	0.5	–
α-Selinene	1,500	1,498	1.4	1.2	2.4	–	–	0.2	1.7	2.4	0.1	–	–
Bicyclogermacrene	1,500	1,500	2.3	2.0	3.8	4.3	13.5	0.1	1.7	–	–	10.7	27
α-Muurolene	1,503	1,500	0.1	0.6	1.3	3.0	4.2	0.4	0.9	1.9	1.0	–	0.5
α-Bulnesene	1,510	1,509	0.2	–	2.1	–	–	–	–	0.4	–	0.7	–
(*E*)-Isoosmorhizole^±^	1,512	1,517	–	–	–	–	–	35.2	–	–	–	–	–
γ-Cadinene	1,517	1,513	0.1	–	–	5.1	5.7	–	1.3	2.5	1.2	0.4	–
δ-Cadinene	1,522	1,522	1.2	–	1.2	–	–	1.0	–	–	–	–	–
Myristicin	1,524	1,518	1.1	–	–	–	–	–	–	–	–	–	–
(*E*) Cadina-1,4-diene	1,527	1,533	–	–	–	4.3	10.1	–	7.1	–	1.3	0.3	21
α-Cadinene	1,537	1,537	–	–	–	–	–	–	–	–	–	–	–
Germacrene B	1,561	1,559	0.2	–	2.1	0.4	1.2	–	0.6	–	–	0.1	–
(*E*)-Nerolidol^S^	1,566	1,561	0.1	–	1.0	5.2	0.6	–	0.9	9.2	–	0.9	–
Palustrol	1,572	1,567	–	–	–	–	0.3	–	0.3	–	–	0.1	0.2
γ-Asarone^S^	1,577	1,572	–	22.0	–	–	–	–	–	–	–	–	–
Spathulenol	1,581	1,577	0.1	–	0.3	0.2	1.9	–	0.8	2.2	0.3	0.7	3.1
(-)-Caryophyllene oxide^S^	1,587	1,582	–	–	0.6	0.8	–	0.1	4.3	1.1	3.4	0.8	0.9
Veridiflorol	1,596	1,592	0.3	–	0.3	0.4	1.5	–	0.3	0.7	0.5	0.6	0.9
Guaiol	1,601	1,600	–	–	–	5.2	–	–	–	1.5	–	–	0.9
Humulene epoxide II	1,613	1,608	–	–	–	–	1.0	–	0.3	0.2	–	–	–
β-Asarone	1,623	1,616	–	–	–	–	–	0.4	–	–	0.3	–	–
Methoxy-4,5-(methylenedioxy)-propiophenone isomer^±^	1,627	1,627	–	–	–	–	–	2.3	–	–	–	–	–
Dillapiole^S^	1,632	1,620	81.0	–	2.2	1.7	–	–	–	–	–	0.1	–
epi-α-Muurolol	1,646	1,640	0.3	–	1.5	1.1	3.2	–	1.1	1.8	0.6	0.6	0.5
Torreyol	1,650	1,644	–	–	0.6	3.3	2.6	–	1.1	0.4	0.3	0.4	0.2
α-Cadinol	1,659	1,652	–	0.1	2.9	–	5.3	–	1.7	3.1	0.9	1.2	0.5
α-Asarona^S^	1,682	1,675	–	–	–	–	–	0.9	–	–	–	–	–
Apiole^S^	1,686	1,677	0.2	–	–	–	–	–	–	–	–	–	–
2-Methoxy-4,5-(methylenedioxy)-propiophenone^±^	1,717	1,713	–	–	–	–	–	4.7	–	–	–	–	–

RI^a^ *, Retention Index calculated against C8-C40 n-alkanes on the HP-5 m column*;

RI^b^ *, Retention index from literature (Adams, 2007); PAD, Piper aduncum; PAR, Piper auritum; PAB, Piper arboreum; PCR, Piper crassinervium; PGA, Piper gaudichaudianum; PMA, Piper marginatum; PHE, Piper hemmendorffii; PAM, Piper amalago; PLB, P. lindbergii; PCE, Piper cernuum; PLU, Piper lucaenum*;

s *Compound identity confirmed with an authentic standard, the remaining compounds were identified by comparing the RI and mass spectra with the Adams and Wiley databases (see text for details)*.

± *Compound IR corresponds to those found for Piper marginatum in Andrade et al. (2008)*.

* *Unidentified compounds*.

### Evaluation of the Larvicidal Activity of the Main Compounds in the EOs

The commercially available compounds *(E)*-Anethole, β-Asarone, (*E*)-β-Caryophyllene, γ-Terpinene, *p*-Cymene, Limonene, α-Pinene and β-Pinene and Dillapiole, obtained by fractionation of EO of *P. aduncum* using flash chromatography were submitted to further evaluation to determine whether they were involved as the active compounds in the EOs. Thus, pure standards were diluted in water and 2% DMSO, in a final volume of 50 mL, to a final concentration of 100 ppm (screening concentration). Out of the nine compounds evaluated, only (*E*)-β-Caryophyllene did not show activity on *A. aegypti* larvae in the three strains at the screening concentration. Nevertheless, the phenylpropanoids (Dillapiole, *(E*)-Anethole and γ-Asarone) and monoterpenes (γ-Terpinene, *p*-Cymene, Limonene, α-Pinene and β-Pinene) showed larvicidal activity in the range of 90–100% (Table 7). Additionally, when comparing the LC_50_ and LC_90_ of the three phenylpropanoids, Dillapiole displayed the lowest LC_50_ for the three strains, followed by *(E*)-Anethole and γ-Asarone (Table 8). Among the five monoterpenes tested, Limonene and γ-Terpinene showed the lowest LC_50_ for the three strains (Table 8).

**Table 7:** Percentage of dead larvae after 24 h of exposure to major compounds of the studied *Piper* species at concentration 100 ppm.

**Major compounds of the studied *Piper* species**	**Strains**		
	**Susceptible**	**Resistant**	
	**Rockfeller**	**Venda Nova**	**Pampulha**
Dillapiole	100	100	100
*E-*Anethole	98.89	96.67	100
γ-Asarone	98.89	96.67	97.78
(*E*)-β-Caryophyllene	0	0	0
γ-Terpinene	94.44	93.3	100
*p*-Cymene	91.1	92.2	90
Limonene	100	100	100
α-Pinene	90	91.1	100
β-Pinene	95.5	97.7	91.1

**Table 8:** Evaluation of lethal concentrations of major compounds in *Aedes aegypti* larvae of resistant and susceptible strains to pyrethroids during 24 h exposure to major compounds of *Piper* species.

**Compounds**	**Strains**	**Slope ± SD**	**LC_**50**_ (ppm) (95% CI)**	**LC_**90**_ (ppm) (95% CI)**
Dillapiole	Rockfeller	2.6 ± 0.2	15.06 (11.94–18.33)	46.16 (35.62–68.71)
	Venda Nova	2.6 ± 0.2	15.75 (12.01–19.74)	47.96 (35.21–77.11)
	Pampulha	2.6 ± 0.1	17.60 (14.24–21.28)	54.56 (41.77–82.48)
*E-*Anethole	Rockfeller	4.0 ± 0.3	34.41 (25.60–42.10)	71.03 (54.85–136.85)
	Venda Nova	4.3 ± 0.3	38.20 (29.33– 47.26)	75.39 (57.74–153.58)
	Pampulha	3.9 ± 0.3	38.98 (33.57–44.52)	82.72 (67.24–120.97)
γ-Asarone	Rockfeller	3.7 ± 0.3	32.65 (29.91–35.20)	71.92 (64.20–83.85)
	Venda Nova	3.3 ± 0.1	37.85 (34.73–40.96)	92.52 (79.59–114.69)
	Pampulha	3.3 ± 0.3	36.23 (31.02–41.20)	88.34 (71.06–130.59)
	Rockfeller	2.7 ± 0.2	25.29 (21.25–29.30)	74.77 (59.26–108.25)
γ-Terpinene	Venda Nova	2.6 ± 0.2	24.58 (21.87–27.26)	76.33 (64.23–96.71)
	Pampulha	2.7 ± 0.2	25.00 (22.41–27.58)	72.55 (61.87–89.92)
	Rockfeller	3.6 ± 0.3	44.80 (41.23–48.48)	100.71 (88.53–119.53)
*p*-Cymene	Venda Nova	3.4 ± 0.3	49.25 (45.30–53.52)	115.51 (99.70–141.21)
	Pampulha	3.5 ± 0.3	47.39 (43.60–51.43)	109.83 (95.47–132.59)
	Rockfeller	3.1 ± 0.2	21.86 (18.43–25.33)	55.02 (45.22–72.76)
Limonene	Venda Nova	3.2 ± 0.2	23.23 (18.76–27.97)	57.94 (45.53–85.24)
	Pampulha	3.0 ± 0.2	21.92 (17.01–27.27)	58.38 (44.19–93.46)
	Rockfeller	4.2 ± 0.2	44.17 (37.68–50.50)	71.92 (74.59–112.61)
α-Pinene	Venda Nova	4.1 ± 0.2	45.17 (37.72–52.43)	92.52 (76.84–124.16)
	Pampulha	3.9 ± 0.2	45.70 (39.48–51.80)	96.49 (82.01–122.45)
β-Pinene	Rockfeller	2.8 ± 0.1	32.97 (26.97–38.92)	93.11 (75.49–126.12)
	Venda Nova	2.8 ± 0.1	33.35 (26.31–40.40)	95.70 (75.13– 138.87)
	Pampulha	2.6 ± 0.1	35.13 (26.02–44.53)	105.59 (78.01–179.01)

*95 % CI, 95% confidence interval; LC_50_, 50% lethal concentration; LC_90_, 90% lethal concentration; SD, standard deviation*.

## Discussion

Studies focusing on the investigation of EOs from plants from the perspective of discovering new ovicides, larvicides, adulticides and repellents have been an important strategy for controlling agricultural pests, vectors of medical-veterinary importance or urban viruses (Santos et al., 2012; Phukerd and Soonwera, 2014; Govindarajan et al., 2016; Benelli et al., 2017; Muturi et al., 2017; Luz et al., 2020a). Regarding the urban diseases in tropical regions, *A. aegypti* is considered one of the main targets since it has great dispersal capacity, is the vector of DENV, ZIKV, CHIKV viruses, and has developed a remarkable resistance to commercially available insecticides (Smith et al., 2016).

The availability of two strains of *A. aegypti* resistant to Deltamethrin (Table 1) prompted us to seek alternatives to control these populations by screening bioactive EOs from plant species. Despite Deltamethrin not having been used to control *A. aegypti* in Brazil, it is a stable molecule with a well-known mechanism of action, and it is a standard pyrethroid in studies with insecticide resistance.

In this article, we adopted the WHO methodology (2005) to perform larvicidal tests against *A. aegypti* (Dias et al., 2015; Luz et al., 2020a). However, as the World Health Organization does not establish criteria to recognize larvicidal activity, in the present study, we choose the level of 90–100% of larvicidal activity for selecting active EOs as previously suggested (Cheng et al., 2003; Dias et al., 2014; Intirach et al., 2016; Muturi et al., 2017). Based on this criterion, EOs from five *Piper* out of 10 species tested showed larvicidal activity (Table 3). The efficiency of EOs from *Piper* species as botanical insecticides against various arthropods, including mosquito larvae of the species *A. aegypti* has been previously demonstrated. For instance, EOs of *P. marginatum* (Autran et al., 2009), *P. aduncum* (de Almeida et al., 2009; Oliveira et al., 2013), *P. gaudichaudianum* (de Morais et al., 2007), *P. arboreum* (Santana et al., 2015), and *P. capitarianum* (França et al., 2021) have displayed an efficient larvicidal action on *A. aegypti*. However, to the best of our knowledge, the present study is the first to demonstrate the bioactivity of EOs of the genus *Piper* and the main active compounds in essential oils in strains of pyrethroid-resistant *A. aegypti* larvae.

Among the five species that showed larvicidal activity against *A. aegypti, P. aduncum* had a lower LC_50_ compared to the other four *Piper* considered active, and previous reports for *P. aduncum* EO activity against larvae of *A. aegypti* led to variable values of LC_50_: 46 ppm (Santana et al., 2015); 50.9 ppm (de Almeida et al., 2009), and up to 289.9 ppm (Oliveira et al., 2013). Our average LC_50_ value of 25 ppm for EO from *P. aduncum* against the resistant strains (VN and PAMP) and SRL, is similar to that described by Scalvenzi et al., 2019 which was 23.73 ppm.

The analysis of the LC_50_ and LC_90_ of the five *Piper* species active against PAMP, VL, and SRL strains (Table 4) indicated comparable LCs values among them, indicating activity of *Piper* sp. EOs regardless of insect resistance to commercial pyrethroids. Such similar larvicidal activity, in populations of *A. aegypti* resistant and susceptible to the organophosphate temephos, was observed with EOs of *Syzygium aromaticum* (Myrtaceae) and *Citrus sinensis* (Rutaceae) (Araújo et al., 2016), while a study of EO from *Petroselinum crispum* (Apiaceae) showed no significant differences of the LC_50_ for EO larvicidal activity against the pyrethroid resistant and susceptible strains of *A. aegypti* (Intirach et al., 2016). Our results agree with previous studies of plant EOs and highlights their potential of acting as efficient larvicides on mosquito strains that are resistant to different types of insecticides, whose use has already led to the development of resistant populations in Brazil (for e.g.: Temephos–Valle et al., 2019; pyrethroids—this study and Costa et al., 2020) and elsewhere.

Although *Piper* species (e.g., *P. hemmendorffii, P. lindbergii, P. amalago, P. cernuum*) did not show any larvicidal activity, in previous studies their major compounds such as α-Pinene (Ali et al., 2014), β-Pinene (Lee and Ahn, 2013; Ali et al., 2014), (*E*)-Nerolidol (Ali et al., 2013), Limonene (Cheng et al., 2013; Lee and Ahn, 2013; Rocha et al., 2015; Nascimento et al., 2017), (*E*)-β-Caryophyllene (Ali et al., 2014, 2015), and β-Myrcene (Cheng et al., 2013; Lee and Ahn, 2013) presented larvicidal activity against *A. aegypti*. This suggests that minor compounds might negatively interfere with oil larvicidal activity, opening new possibilities to study synergisms between compounds, as their interactions are long-lasting and complex, especially because minor compounds often present biological effects.

Out of the major phenylpropanoid compounds tested, Dillapiole, (*E*)*-*Anethole and γ-Asarone (Table 7), only (*E*)-Anethole was previously reported as an active compound in essential oils against *A. aegypti* larvae. The LC_50_ interval found to (*E*)-anethole (28.0–30.6 ppm) (Rocha et al., 2015), overlaps the confidence interval found for the three strains in the present study (25.60–44.52 ppm) (Table 8). However, in a study carried out by Pandiyan et al. (2019) the LC_50_ confidence interval, was 48.89–51.50 ppm for the (*E*)*-*anethole, which is a higher value than that found in our study.

The sesquiterpene (*E*)-β-Caryophyllene was the only compound that did not show potential larvicidal activity (Table 7). In fact, this data is in accordance with that found by Luz et al. (2020b), but different to the LC_50_ values found by Ali et al. (2014) (26, μg/mL), Lee and Ahn (2013) (38.58 μg/mL), and Borrero-Landazabal et al. (2020) (29.28 μg/mL).

Larvae treated with *Piper* EOs that showed larvicidal activity, were completely damaged, compared with control groups, particularly in the chest and segments of the abdominal region. Specifically, the midgut region was destroyed, and content became dark. These visual observations after the exposure period to *Piper's* EOs or to major active compounds, indicate morphological (structural) changes in the larva. Therefore, the similar values of LCs in resistant and susceptible strains suggest a mode of action unrelated to the known biochemical and target site mutations in resistant strains.

The chemical profile of the compounds described in the EOs of the *Piper* species investigated here (Table 6) has already been described in previous studies. For instance, Dillapiole is a typical compound for *P. aduncum* (Pino et al., 2004; de Almeida et al., 2009; Guerrini et al., 2009; Volpe et al., 2016; Scalvenzi et al., 2019); Germacrene D for *P. arboreum* (Machado et al., 1994; Mundina et al., 1998; Navickiene et al., 2006; Perigo et al., 2016; Santana et al., 2016); α-Pinene, β-Pinene, and (*E*)-β-Caryophyllene for *P. crassinervium* (Morandim et al., 2010; Morandim-Giannetti et al., 2010; Perigo et al., 2016); α-Humulene and Bicyclogermacrene for *P. gaudichaudianum* (Von Poser et al., 1994; Andrade et al., 1998; Morandim-Giannetti et al., 2010; Sperotto et al., 2013); α-Pinene for *P. amalago* (Potzernheim et al., 2006; Perigo et al., 2016) and *P. cernuum* (Bernuci et al., 2016; Perigo et al., 2016).

In case of *P. lucaenum*, the major compound Bicyclogermacrene described in our study was replaced by α-pinene in another study (Marques et al., 2015). In fact, large chemical variability in EOs of *Piper* species has already been reported (Andrade et al., 2008). For instance, EOs from 22 samples of *P. marginatum* leaves collected in different areas and ecosystems of the Brazilian Amazon, separated by up to 1000 km, exhibited different major compounds depending on the place of origin. In our study, while the species *P. marginatum* had (*E*)-Anethole as a major compound, analysis of other specimens led to the characterization of 3,4-methylenedioxy propiophenone (Macêdo et al., 2020), and (*Z*)- or (*E*)-Asarone, and Patchouli alcohol (Autran et al., 2009) as major compounds. Such variability can result from different environmental conditions, soil composition, development, biotic factors, and plant genetic diversity (Gobbo-Neto and Lopes, 2007; Silva et al., 2019; Mollaei et al., 2020).

## Conclusion

Our results suggest the promising role of the EOs of these five species of *Piper* as an alternative in controlling *A. aegypti* mosquito larvae of susceptible and insecticide resistant strains. The efficacy of these EOs suggest their use as alternative bioinsecticides in the management of insecticide resistant mosquitoes. Despite the ease of obtaining EOs by hydrodistillation, which is an advantage together the green appeal of such products, their high chemical variability may represent a potential drawback for product development unless a rigorous cultivation control or full understanding of the regulatory processes in the biosynthesis of these phenylpropanoids are achieved.

## Data Availability Statement

The original contributions presented in the study are included in the article/Supplementary Material, further inquiries can be directed to the corresponding author.

## Author Contributions

AP, MK, and GP designed the research carried. AP, MK, GP, LY, and MS interpreted the data and contributed to writing the manuscript. AS, RP, and WN contributed to methodology and investigation. All authors contributed to the article and approved the submitted version.

## Conflict of Interest

The authors declare that the research was conducted in the absence of any commercial or financial relationships that could be construed as a potential conflict of interest.
