# Supplementary material for: Larvicidal Activity of Essential Oils From Piper Species Against Strains of Aedes aegypti (Diptera: Culicidae) Resistant to Pyrethroids
Source: Front Plant Sci. 2021 Jun 4;12:685864. doi: 10.3389/fpls.2021.685864 (PMC8213341; doi:10.3389/fpls.2021.685864)
Supplement: Supplementary file 1 [file Data_Sheet_1.doc]

Supplementary Material

**List of Figures**

# Figure S1. GC-MS Chromatograms of the Essential Oils (EOs) of *Piper aduncum, P. amalago, P. arboreum* and *P. lindbergii.* Numbers show EO compounds selected for single compound larvicidal assays with insecticide resistant and susceptible strains of *Aedes aegypti* larvae. 1: α-Pinene, 2: *p*-Cymene, 3: Limonene, 4: δ-Elemene, 5: α-Copaene, 6: (*E*)-β-Caryophyllene, 7: Germacrene D, 8: Dillapiole.

**Figure S2.** GC-MS Chromatograms of the Essential Oils (EOs) of *Piper cernuum, P. crassinervium, P. gaudichaudianum* and *P. hemmendorffii*. Numbers show EO compounds selected for single compound larvicidal assays with insecticide resistant and susceptible strains of *Aedes aegypti* larvae. **1**: α-Pinene, **2**: β-Pinene, **3**: Limonene, **4**: (*E*)-β-Caryophyllene, **5**: α-Humulene, **6**: Bicyclogermacrene.

**Figure S3.** GC-MS Chromatograms of the Essential Oils (EOs) of *Piper lucaenum* and *P. marginatum*. Numbers show EO compounds selected for single compound larvicidal assays with insecticide resistant and susceptible strains of *Aedes aegypti* larvae. **1**: α-Pinene, **2**: Myrcene, **3**: (*E*)*-*Anethole, **4**: Bicyclogermacrene, **5**: (*E*)*-*Isoosmorhizole, **6**: (*E*)*-*Isoosmorhizole.

**Figure S4.** Light micrographs of *Aedes aegypti* larvae from strains that are resistant (Venda Nova and Pampulha) or susceptible (Rockfeller) to pyrethroids. The morphology of the whole body of the larvae was observed with a stereo microscope (4× magnification). Control and vehicle control larvae showed normal appearances of typical structures, with a distinguished head, thorax, and abdominal regions. Larvae treated with *Piper* EOs (exemplified by *P. aduncum*), and some major compounds (Dillapiole, (*E*)-Anethole and α-Pinene) that showed larvicidal activity, were completely damaged as compared with control groups, particularly in the thorax and in segments of the abdominal region. Specifically, the midgut region was ruptured, and content became dark.

**Figure S1.** GC-MS Chromatograms of the Essential Oils (EOs) of *Piper aduncum, P. amalago, P. arboreum* and *P. lindbergii*. Numbers show EO compounds selected for single compound larvicidal assays with insecticide resistant and susceptible strains of *Aedes aegypti* larvae. **1**: α-Pinene, **2**: *p*-Cymene, **3**: Limonene, **4**: δ-Elemene, **5**: α-Copaene, **6**: (*E*)-β-Caryophyllene, **7**: Germacrene D, **8**: Dillapiole.

**Figure S2.** GC-MS Chromatograms of the Essential Oils (EOs) of *Piper cernuum, P. crassinervium, P. gaudichaudianum* and *P. hemmendorffii*. Numbers show EO compounds selected for single compound larvicidal assays with insecticide resistant and susceptible strains of *Aedes aegypti* larvae. **1**: α-Pinene, **2**: β-Pinene, **3**: Limonene, **4**: (*E*)-β-Caryophyllene, **5**: α-Humulene, **6**: Bicyclogermacrene.

**
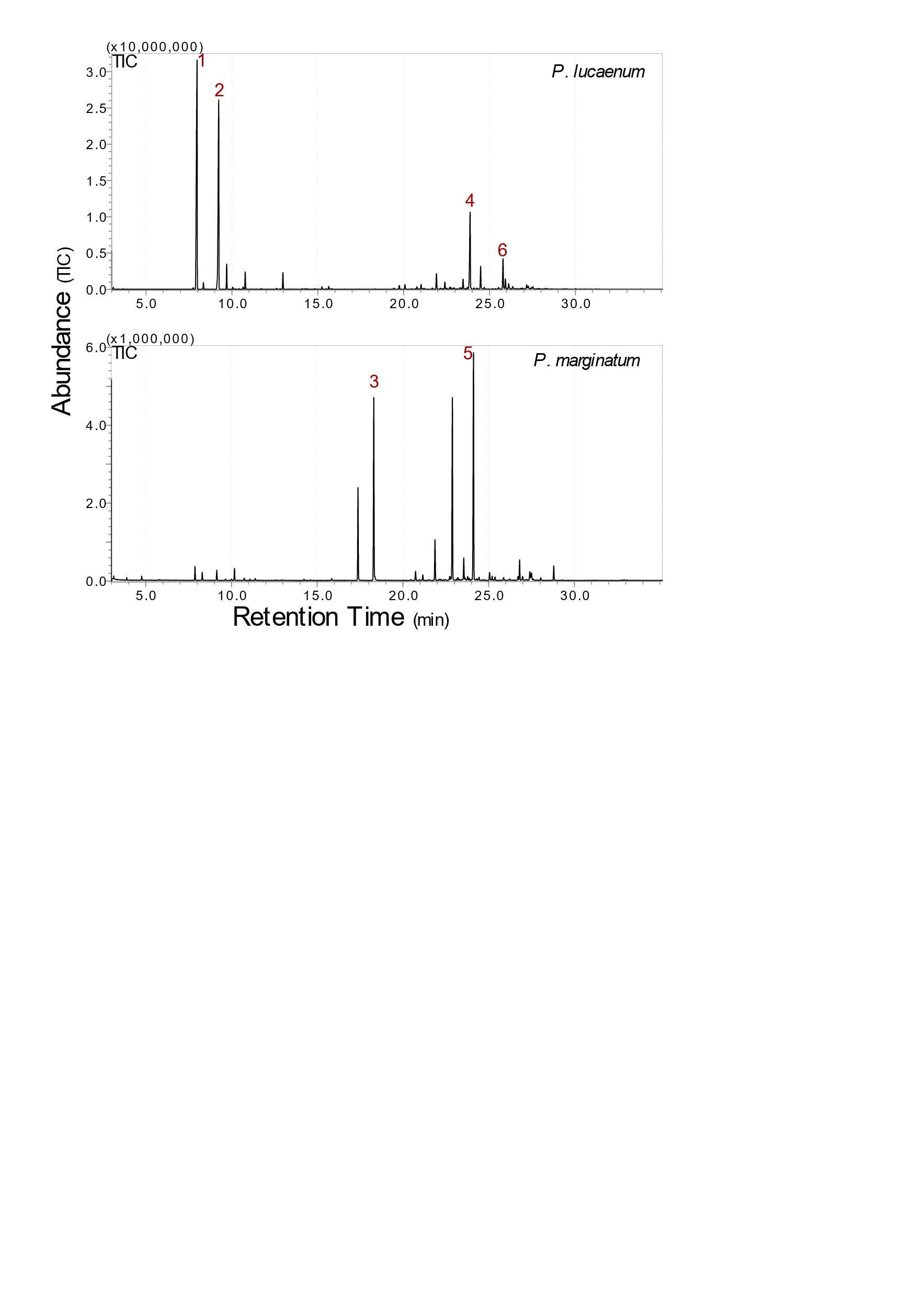
**

**Figure S3.** GC-MS Chromatograms of the Essential Oils (EOs) of *Piper lucaenum* and *P. marginatum*. Numbers show EO compounds selected for single compound larvicidal assays with insecticide resistant and susceptible strains of *Aedes aegypti* larvae. **1**: α-Pinene, **2**: Myrcene, **3**: (*E*)*-*Anethole, **4**: Bicyclogermacrene, **5**: (*E*)*-*Isoosmorhizole, **6**: (*E*)*-*Isoosmorhizole.

**Figure S4.** Light micrographs of *Aedes aegypti* larvae from strains that are resistant (Venda Nova and Pampulha) or susceptible (Rockfeller) to pyrethroids. The morphology of the whole body of the larvae was observed with a stereo microscope (4× magnification). Control and vehicle control larvae showed normal appearances of typical structures, with a distinguished head, thorax, and abdominal regions. Larvae treated with *Piper* EOs (exemplified by *P. aduncum*), and some major compounds (Dillapiole, (*E*)-Anethole and α-Pinene) that showed larvicidal activity, were completely damaged as compared with control groups, particularly in the thorax and in segments of the abdominal region. Specifically, the midgut region was ruptured, and content became dark.
